# Functional Neuroimaging Correlates of Autobiographical Memory Deficits in Subjects at Risk for Depression

**DOI:** 10.3390/brainsci5020144

**Published:** 2015-04-24

**Authors:** Kymberly D. Young, Patrick S. F. Bellgowan, Jerzy Bodurka, Wayne C. Drevets

**Affiliations:** 1 Laureate Institute for Brain Research, 6655 South Yale Avenue, Tulsa, OK 74136, USA; E-Mails: jbodurka@laureateinstitute.org (J.B.); wdrevets@ITS.JNJ.com (W.C.D.); 2 National Institute of Neurological Disorders and Stroke, National Institutes of Health, Bethesda, MD 20892, USA; E-Mail: patrick.frostbellgowan@nih.gov; 3 Biomedical Engineering Center, College of Engineering, University of Oklahoma, Norman, OK 73019, USA; 4 Janssen Research and Development, LLC, of Johnson & Johnson, Inc., Titusville, NJ 08560, USA

**Keywords:** autobiographical memory, depression, remitted depression, fMRI, hereditary risk for depression, prefrontal cortex

## Abstract

Overgeneral autobiographical memory (AM) manifests in individuals with major depressive disorder (MDD) tested during depressed (dMDD) or remitted phases (rMDD), and healthy individuals at high-risk (HR) for developing MDD. The current study aimed to elucidate differences in hemodynamic correlates of AM recall between rMDDs, HRs, and controls (HCs) to identify neural changes following previous depressive episodes without the confound of current depressed mood. HCs, HRs, and unmedicated rMDDs (*n* = 20/group) underwent fMRI while recalling AMs in response to emotionally valenced cue words. HRs and rMDDs recalled fewer specific and more categorical AMs relative to HCs. During specific AM recall, HRs had increased activity relative to rMDDs and HCs in left ventrolateral prefrontal cortex (VLPFC) and lateral orbitofrontal cortex. During positive specific AM recall, HRs and HCs had increased activity relative to rMDDs in bilateral dorsomedial prefrontal cortex (DMPFC) and left precuneus. During negative specific AM recall HRs and HCs had increased activity in left VLPFC and right DMPFC, while rMDDs had increased activity relative to HRs and HCs in right DLPFC and precuneus. Differential recruitment of medial prefrontal regions implicated in emotional control suggests experiencing a depressive episode may consequently reduce one’s ability to regulate emotional responses during AM recall.

## 1. Introduction

It is a well established and replicated finding in the literature that patients with major depressive disorder (MDD), when compared to healthy controls, recall fewer *specific* autobiographical memories (AMs), and instead recall more overgeneral categorical AMs [1]. Specific AMs are operationally defined as memories of an event that occurred at an identifiable time and location and did not last longer than 24 h (*i.e.*, my 18th birthday party), whereas categorical AMs are more general memories of recurring events without reference to a single occurrence (*i.e.*, I go to the grocery store every Sunday) [1]. This abnormality persists despite remission of depressive symptoms [2,3,4] and is present in individuals at high-risk (HR) for developing MDD based on having a first degree relative diagnosed with the disorder [5]. These findings suggest that AM recall constitutes a trait-like marker of MDD.

The neurobiological underpinnings of altered AM recall in depression are just beginning to be explored. Recent functional magnetic resonance imaging (fMRI) studies in our laboratory demonstrated that currently depressed subjects (dMDD) [6], otherwise healthy individuals at high hereditary risk for MDD [5], and individuals remitted from MDD (rMDD) [7] all manifest differences relative to healthy controls (HCs) in the regional hemodynamic activity observed during specific AM recall. The HRs relative to HCs and dMDDs showed decreased blood-oxygen-level-dependent (BOLD) activity in prefrontal regions involved in ruminative processes and self-focus including the frontal operculum, pregenual anterior cingulate cortex (ACC), and frontal polar cortex [5]. The dMDDs relative to HCs exhibited decreased activity in medial temporal and prefrontal regions that form part of the core set of brain regions recruited during AM [8], including the hippocampus and ACC [6]. The rMDDs relative to HCs and dMDDs exhibited increased activity in regions involved in emotional control including dorsomedial prefrontal cortex (DMPFC) and orbitofrontal cortex (OFC), as well as in core AM regions including the hippocampus and middle temporal gyrus [7]. A recent study by Hach *et al.* (2014) also found decreased hippocampal, as well as precuneus and posterior cingulate activation, and increased inferior frontal gyrus and frontal polar regions in dMDD participants relative to controls during specific AM recall [9]. Other studies examining AM *recognition* (as opposed to AM recall) in dMDD have found increased dorsolateral prefrontal cortex (DLPFC) activity during negative AM recognition [10,11], and increased activity in ventrolateral prefrontal cortex (VLPFC) during positive AM recognition [10] in dMDD relative to HC participants. Collectively, these data suggests that when depressed individuals are able to recall specific AMs, they show blunted activity in core regions involved in AM recall [8] relative to healthy controls, an abnormality which could either cause or reflect the impairment in recalling specific AMs which these individuals manifest. Furthermore, individuals at risk for experiencing a major depressive episode (MDE) but who are currently not experiencing any significant mood pathology engage regions during specific AM recall that putatively are involved in reducing rumination and self-focus and enhancing emotional control.

The current study aimed to extend findings of altered hemodynamic activity during AM recall in mood disorders [5,7], by directly comparing our previous HR and rMDD participants and examining more specifically the hemodynamic correlates of positively *versus* negatively valenced AMs. In doing so we controlled for vulnerability to MDD, as both groups were at increased risk for developing an MDE based either upon having a first-degree relative with the disorder or on having experienced an MDE previously. An important difference between HR and rMDD participants is that the rMDD subjects have experienced an MDE in the past. Therefore, the hemodynamic differences between these groups may reveal either pathophysiological or adaptive changes in neural function that resulted from experiencing depressive episodes, which presumably would be distinct from the functional anatomical correlates associated with the risk for depression. Crucially, in contrast to our previous studies which included currently depressed MDD participants, the groups studied herein are unmedicated and euthymic, and the direct comparison of these groups avoids potential confounds of mood state and nonspecific effects of illness (e.g., sleep deprivation) or treatment [12,13,14]. Thus, research which illuminates differences between these groups may guide the development of new approaches to prevent an MDE. While the HR and MDD participants are the same as those included in our previous studies, we recruited an additional 20 HCs. This new sample of HCs ensures that our results are not biased by using the same noise distribution of the original HC sample

Based on the results of past studies examining the hemodynamic correlates of AM recall in depression [5,7], we predicted that the HR and rMDD participants would exhibit differences in prefrontal brain regions involved in self-referential processing, rumination, and emotional control. More specifically, we predicted that rMDD participants would manifest higher activity than HRs and HCs in regions where we previously found increased activity in dMDD participants compared to HRs, including the ACC, VLPFC and medial PFC. These predictions were based on the hypothesis that the behavioral and functional anatomical abnormalities evident during AM recall in currently depressed MDD subjects persist into remission, as well as the results from our previous studies using a subset of the current participants [5,7].

## 2. Results

### 2.1. Behavioral Results

Participant characteristics and general task performance data during fMRI scanning are presented in Table 1. The groups did not differ in their mean age or IQ (*Fs*(2,57) < 0.04, *ps* > 0.96), or in their self-report ratings (or pre to post-scan change) on the POMS and STAI (*Fs*(2,57) < 1.84, *ps* > 0.17).There was a significant difference on the clinician administered symptom ratings (*Fs*(2,57) > 3.95, *ps* < 0.02). While rMDD participants had scores within the non-depressed range on the MADRS and HDRS, these scores nevertheless were higher compared to the HRs (*ts*(38) > 2.68, *ps* < 0.01), and HCs (*ts*(38) > 2.43, *ps* < 0.02), while HRs and HCs did not differ from each other (*ts*(38) < 1.10, *ps* > 0.28). There was no significant difference between groups in performance accuracy on the riser detection task, the number of examples generated for the distinctly valenced categories, or the ease with which such examples were generated (*Fs*(2,57) < 1.62, *ps* > 0.21).

**Table 1:** Participant characteristics and task performance by diagnostic group shown as mean values (with standard deviation).

Demographics	HC	HR	rMDD
N (number females)	20 [13]	20 [14]	20 [12]
Age in years	29 (9)	30 (9)	30 (12)
IQ (as determined by the WASI)	109 (13.9)	109 (8.11)	109 (9.58)
HDRS	0.81 (2.05)	0.75 (1.48)	3.40 (4.17) *, #
MADRS	0.79 (2.08)	0.95 (2.35)	4.55 (4.98) *, #
POMS Total Mood Disturbance	−30.3 (19.9)	−33.7 (15.5)	−28.3 (16.8)
State Anxiety	24.9 (4.17)	26.7 (6.02)	28.3 (6.16)
Trait Anxiety	27.0 (5.09)	26.9 (3.73)	32.9 (7.02)
Pre-Post scan Change in POMS	3.95 (8.84)	3.15 (10.4)	5.60 (19.1)
Pre-post scan change in State Anxiety	0.85 (3.92)	0.50 (5.38)	0.85 (5.69)
Pre-post scan change in Trait Anxiety	−1.35 (3.08)	−1.50 (2.04)	-2.15 (2.83)
**Control Tasks**			
Percent correct—Riser detection task	78.3 (11.5)	74.7 (11.1)	75.7 (10.4)
Number of Examples Positive Categories	5.95 (0.99)	5.75 (1.03)	5.47 (0.81)
Number of Examples Negative Categories	5.64 (1.25)	5.36 (0.91)	5.05 (0.91)
Number of Examples Neutral Categories	6.16 (0.83)	6.14 (0.82)	5.83 (0.70)
Percent of Positive Examples Easy to Generate	87.8 (12.3)	84.7 (13.1)	85.8 (26.0)
Percent of Negative Examples Easy to Generate	83.3 (18.8)	77.1 (19.9)	79.1 (19.8)
Percent of Neutral Examples Easy to Generate	83.5 (22.1)	86.3 (10.4)	83.5 (20.4)
**Memory Type (% recalled)**			
Specific	65.0 (10.9)	48.5 (13.9) *	42.2 (10.9) *
Categorical	16.5 (6.56)	28.0 (11.4) *	32.9 (9.99) *
Extended	3.14 (2.64)	5.12 (3.52)	4.12 (3.12)
Semantic	4.43 (3.36)	7.01 (5.18)	6.85 (5.62)
No Memory	1.93 (2.48)	2.08 (3.58)	3.31 (4.31)
Can’t Recall Post Scan	9.25 (6.67)	9.45 (5.85)	10.5 (7.43)

Numbers in parentheses indicate one standard deviation of the mean. * Indicates a significant difference from the HC group at *p*_corrected_ < 0.05; ^#^ Indicates a significant difference from the HR group at *p*_corrected_ < 0.05. Abbreviations: HC = healthy control; HR = high-risk; HDRS = Hamilton Rating Scale for Depression; MADRS Montgomery-Asberg Depression Rating Scale; rMDD = remitted major depressive disorder; POMS = Profile of Mood States; WASI = Wechsler Abbreviated Scale of Intelligence.

Importantly, there was a significant Group by Memory Type interaction indicating group difference in the percent of specific and categorical AMs recalled (*Fs*(2,57) > 15.7, *ps* < 0.001). In contrast, the groups did not differ in the percent of extended, semantic, no memory or can’t recall responses (*Fs*(2,57) < 1.80, *ps* > 0.17). A priori contrasts showed that while HCs recalled more specific and fewer categorical AMs than both MDD (*ts*(38) > 6.63, *ps* < 0.01) and HR participants (*ts*(38) > 4.19, *ps* < 0.001), the HR and MDD groups did not differ from each other on the percent of specific or categorical AMs recalled (*ts*(38) < 1.46, *ps* > 0.15). Properties of specific and categorical memory recall are presented in the Supplement Information (Table S1). No significant interaction with the variable Diagnosis was evident (*Fs*(4,114 or 8,110 <1.46, *ps* > 0.22) for the variables arousal, vividness, or age when memory occurred. There was a Diagnosis × Specificity × Valence interaction (*Fs*(4,114) = 9.11, *p* < 0.001), with HCs reporting more specific positive and fewer specific negative AMs than HRs or MDDs (*ts*(38) > 2.15, *ps* < 0.04), while HRs and MDDs did not differ significantly from each other (*ts*(38) < 1.03, *ps* > 0.31). No group difference was found for Specific Neutral AMs or for Categorical AMs of any valence (*ts*(38) < 1.23, *ps* > 0.23).

### 2.2. Functional MRI Results

Table 2 lists regions where BOLD activity increased across the entire sample (all three groups combined), and Table 3 lists group differences in regional BOLD activity for the contrasts performed. When comparing Example Generation to Riser Baseline, in the entire sample BOLD activity increased in left DLPFC, posterior cingulate cortex (PCC), superior temporal and middle temporal gyrus, and bilateral DMPFC and parahippocampus/hippocampus. No significant group difference in regional BOLD activity was identified for this contrast.

**Table 2:** Increases in hemodynamic activity for the different contrasts across all three participant groups.

Contrast	Specific Memories *vs.* Example Generation		Categorical Memories *vs.* Example Generation		Example Generation *vs*. Riser Baseline	
Area	Cluster Size	x, y, z	Cluster Size	x, y, z	Cluster Size	x, y, z
L DLPFC	503	−39, 3, 50	94	45, 11, 46	225	35, −21, 60
R DLPFC	227	39, 5, 48	84	−41, 11, 50		
L DMPFC					37	−9, 11, 60
R DMPFC					44	11, 53, 34
R VLPFC	515	59, 19, 14	173	57, 23, 10		
R OFC			65	53, 21, 4		
L Medial PFC	7809	−1, 59, 34	5123	−1, 59, −2		
R Postcentral G/BA 3	35	21, −35, 56				
L PCC			154	−1, −27, 32	714	−3, −37, 32
L Superior Temporal G	2351	−49, −59, 20			91	65, −41, 12
R Superior Temporal G	2690	21, 13, −36				
L Middle Temporal G	2291	−55, −3, −12	1251	−63, −11, −8	492	−47, −71, 28
R Middle Temporal G	2895	53, −9, −14	1238	61, −1, −12		
L Hippocampus/Parahippocampus	98	−21, −21, −12			4221	−30, −24, −9
R Hippocampus/Parahippocampus	61	17, −11, −24			815	19, −19, −14
L Precuneus	1676	−33, −81, 34	3846	−33, −81, 34		
R Precuneus	1698	45, −71, 36	989	45, −73, 34		
L Cuneus	5364	−1, −67, 32	3631	−1, −65, 32		

Coordinates correspond to the stereotaxic array of Talairach and Tournoux [15], and denote the distance in mm from the origin (anterior commissure), with positive *x* indicating right, positive *y* indicating anterior, and positive *z* indicating dorsal. Cluster size refers to the number of contiguous voxels for which the voxel *t* value corresponds to *p*_corrected_ < 0.05. For all reported clusters, *t* > 3.46. Abbreviations: BA = Brodmann area; DLPFC = dorsolateral PFC; DMPFC = dorsomedial PFC; G = gyrus; L = left; OFC = orbitofrontal cortex; PCC = posterior cingulate cortex; PFC = prefrontal cortex; R = right, VLPFC = ventrolateral PFC.

When comparing Categorical Memory recall to Example Generation, BOLD activity in the entire sample increased in left medial PFC, PCC, and cuneus, right VLPFC, and OFC, and bilateral DLPFC, middle temporal gyrus, and precuneus. No significant group difference in regional BOLD activity was identified for this contrast.

**Table 3:** Regions where hemodynamic activity differed significantly between the diagnostic groups for the different contrasts.

Area	Cluster Size	x, y, z	*F* value	β Weight		
				HC	HR	rMDD
**Specific AMs *vs.* Example Generation**						
HR > HC > rMDD						
L VLPFC	30	−31, 51, 2	6.96	0.17 (0.02)	0.20 (0.03)	−0.04 (0.04)
L Lateral OFC/BA 47	33	−33, 17, −16	7.94	0.21 (0.04)	0.26 (0.03)	0.02 (0.02)
**Positive Specific AMs v Positive Example Generation**						
HR = HC > rMDD						
L DMPFC/BA 9	62	−1, 39, 34	8.51	0.24 (0.03)	0.22 (0.06)	0.02 (0.02)
R DMPFC/BA 9	44	5, 57, 28	7.32	0.38 (0.05)	0.37 (0.07)	0.07 (0.04)
L Precuneus	32	−7, −79, 44	7.51	0.29 (0.04)	0.26 (0.17)	0.05 (0.05)
**Negative Specific AMs v Negative Example Generation**						
HR > HC > rMDD						
L VLPFC	37	−31, 47, 0	9.34	0.11 (0.03)	0.21 (0.06)	0.02 (0.02)
R DMPFC/BA 9	30	9, 41, 28	8.55	0.08 (0.01)	0.14 (0.04)	0.01 (0.02)
rMDD > HR = HC						
R DLPFC	36	25, 7, 34	9.01	−0.04 (0.03)	−0.03 (0.02)	0.05 (0.02)
R Precuneus	35	15, −67, 42	8.21	−0.38 (0.10)	−0.42 (0.06)	0.17 (0.04)
**Categorical AMs *vs.* Example Generation**						
no significant clusters						
**Example Generation *vs.* Riser Baseline**						
no significant clusters						

Coordinates correspond to the stereotaxic array of Talairach and Tournoux [15]. Cluster size refers to the number of contiguous voxels for which the voxel *t* value corresponds to *p*_corrected_ < 0.05. Numbers in parentheses indicate on standard deviation of the mean. Abbreviations: BA = Brodmann area; DLPFC = dorsolateral prefrontal cortex; DMPFC = dorsomedial prefrontal cortex; G = gyrus; HC = healthy control; HR = high-risk; L = left; OFC = orbitofrontal cortex; R = right; rMDD = remitted major depressive disorder; VLPFC = ventrolateral prefrontal cortex.

**Figure 1:**
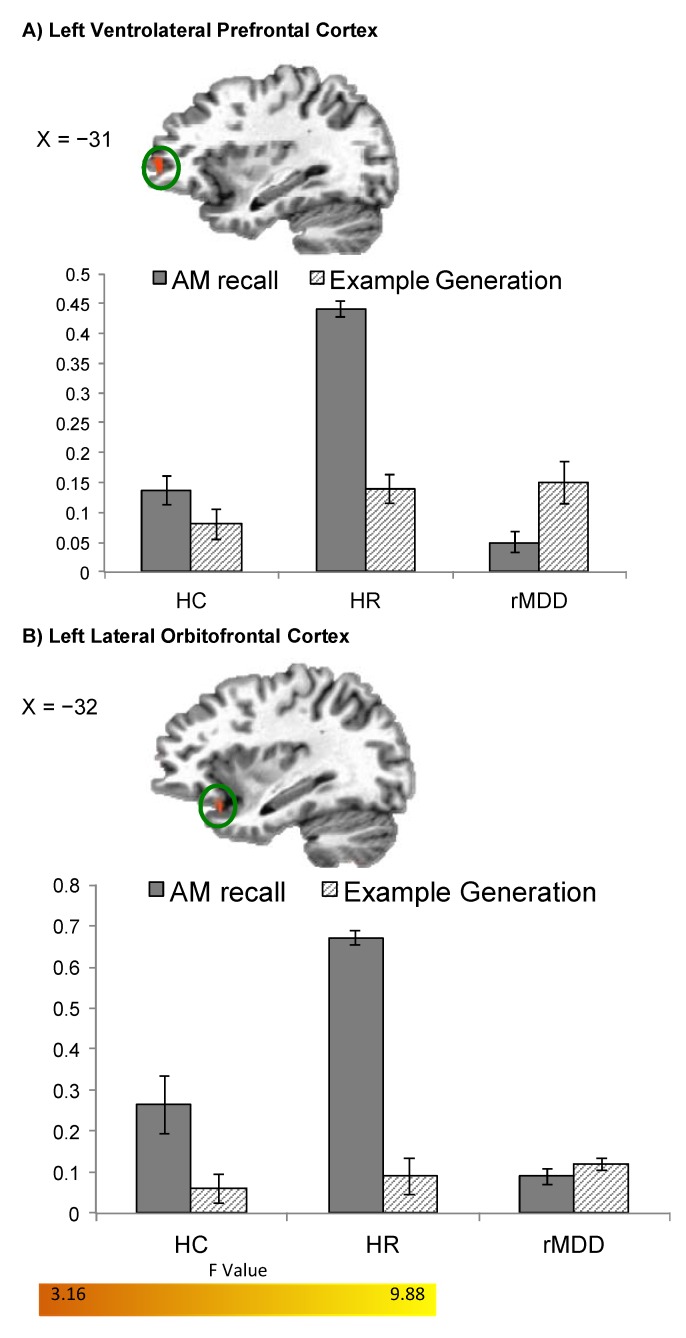
Differences in BOLD activity between HC, HR, and rMDD groups during *specific* AM recall. Regions (**A**) left ventrolateral PFC and (**B**) left lateral orbitofrontal cortex are shown where group BOLD activity differed during *Specific* AM recall *versus* Example Generation (*p*_corrected_ < 0.05). Using the significant clusters in Table 3 as ROIs, beta weights were extracted for the *Specific AMs versus* Riser Baseline conditions, and for the Example Generation *versus* Riser Baseline conditions. Error bars indicate +/− one standard error of the mean. Abbreviations: AM=autobiographical memory; BOLD=blood-oxygen-level-dependent; HC = healthy control; HR = high-risk; rMDD = remitted major depressive disorder; PFC = prefrontal cortex.

When comparing Specific Memory recall to Example Generation, BOLD activity increased in the entire sample in left medial PFC and cuneus, right VLPFC and postcentral gyrus, and bilateral DLPFC, superior temporal gyrus, middle temporal gyrus, hippocampus/parahippocampus, and precuneus. Group differences were evident in the left VLPFC and lateral OFC/BA47 such that HR participants had significantly increased BOLD activity in these regions relative to both HC and rMDD participants (Figure 1).

**Figure 2:**
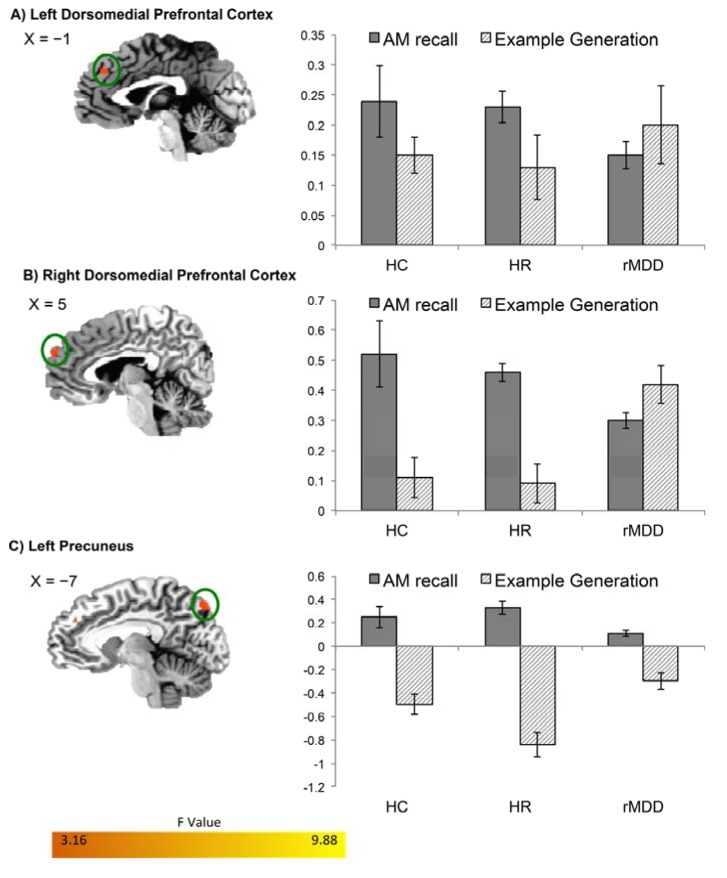
Differences in BOLD activity between HC, HR, and rMDD groups during *positive specific* AM recall. Regions (**A**) left and (**B**) right dorsomedial PFC and (**C**) left precuneus are shown where groups had differential BOLD activity during *Positive Specific* AM recall *versus* Positive Example Generation (p_corrected_ < 0.05). Using the significant clusters in Table 3 as ROIs, beta weights were extracted for the *Positive Specific AMs versus* Riser Baseline conditions, and for the Positive Example Generation *versus* Riser Baseline conditions. Error bars indicate +/− one standard error of the mean. Abbreviations defined in the legend for Figure 1.

**Figure 3:**
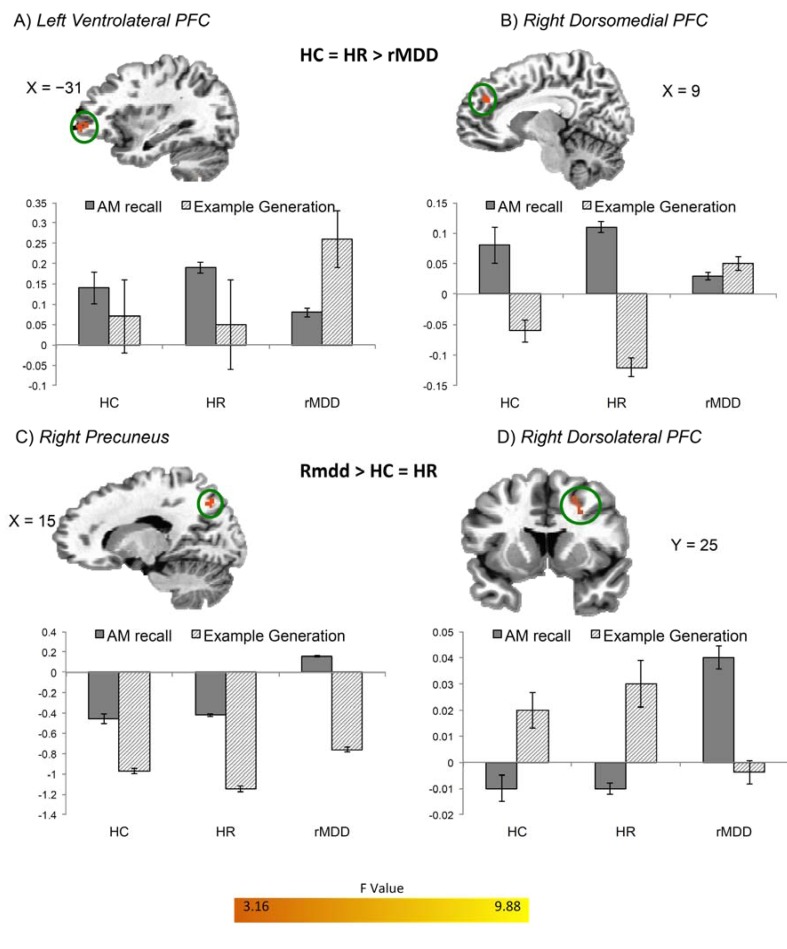
Differences in BOLD activity between HC, HR, and rMDD groups during *negative specific* AM recall. Regions (**A**) left ventrolateral PFC; (**B**) right dorsomedial PFC; (**C**) right precuneus; and (**D**) right dorsolateral PFC are shown where groups had differential BOLD activity during *Negative Specific* AM recall *versus* Negative Example Generation (p_corrected_ < 0.05). Using the significant clusters in Table 3 as ROIs, beta weights were extracted for the *Negative Specific AMs versus* Riser Baseline conditions, and for the Negative Example Generation *versus* Riser Baseline conditions. Error bars indicate +/− one standard error of the mean. Abbreviations defined in the legend for Figure 1.

Group differences in BOLD activity were also evident in the additional contrasts comparing Positive Specific Recall *versus* Positive Example Generation and Negative Specific Recall *versus* Negative Example Generation. During positive AM recall *versus* positive example generation both HC and HR participants showed increased BOLD activity relative to rMDD participants in bilateral DMPFC/BA9 and left precuneus (Figure 2). During negative AM recall *versus* negative example generation (Figure 3) HC and HR participants had increased BOLD activity relative to rMDD participants in right DMPFC/BA9 and left VLPFC, while the rMDD participants had increased BOLD activity relative to HC and HR participants in right DLPFC and precuneus for this contrast.

## 3. Discussion

The current study compared the functional neuroanatomical correlates of AM recall between HC, HR, and rMDD participants. This is the first study to directly compare fMRI data during specific AM recall between HR and rMDD samples. Behaviorally, HCs recalled more specific and fewer categorical AMs compared to both the HR and rMDD groups, who recalled comparable numbers of specific and categorical AMs to each other. That both HR and rMDD participants exhibit the same behavioral pattern of AM recall suggests that AM overgenerality is a trait-like marker of MDD. Despite similar behavioral performance, however, the two participant groups exhibited differences in the brain regions recruited during AM recall. These differences were limited to specific AM recall, however. No difference between groups was found for categorical AM recall, suggesting that the abnormalities associated with vulnerability to MDEs is selective for specific AM processing.

During specific AM recall, HR participants showed increased BOLD activity relative to both HC and rMDD participants in the left VLPFC and lateral OFC. These regions have been implicated in emotional regulation, and abnormalities in structure and/or function have been found in portions of these structures in MDD patients [16]. The VLPFC has been implicated in controlling attention and reducing the influence of distractors [17]. Particularly relevant for the current study, research has implicated the VLPFC in the ability to resolve negative feelings brought on by social exclusion or pain by directing attention to helpful solutions rather than to rumination [18]. The greater activity in this region in the HR participants relative to HC and rMDD participants during specific and *negative* specific AM recall suggests the hypothesis that the HR participants activate this region to regulate or suppress rumination, especially on negative memories, and that this compensatory process may require greater neural activity than in HCs in order to maintain euthymia, as reflected by higher BOLD activity in HRs than HCs.

The lateral OFC is involved modulating responses involved in reward-directed behavior [19]. This region is also involved in the regulation of emotional responses via its influence on amygdala activity, as amygdala and lateral OFC activity are inversely correlated in MDD patients [20,21]. Activation of this region may therefore reflect attempts to attenuate emotional expression or ruminative thoughts, and dysfunction here may contribute to the development of excessive emotional responses and ruminations [21]. Both the OFC and VLPFC are increased in currently depressed MDD relative to control subjects [21,22], and activity increases in healthy subjects during induced sadness [23]. However, activity in both of these regions is inversely correlated with depressive symptom severity [24], and it is noteworthy that successful cognitive behavioral therapy increases the function of these regions [25], and that deep transcranial magnetic stimulation applied over these regions exerts an antidepressant effect in MDD [26,27]. Thus greater activity in the left lateral OFC and VLPFC in general appears to subserve an adaptive or compensatory role in MDD [16].

In contrast, a region in which both HR and HC participants showed increased activity compared to rMDD participants is the DMPFC. In healthy humans activity in this region increases during tasks eliciting emotional responses and evaluations [28], and it is hypothesized that this region is associated with cognitive control processes that serve to regulate emotional responses [29]. Reduced BOLD activity and gray matter volume in the DMPFC has been reported in MDD [30,31,32], and increased activity in this region was associated with better treatment response in MDD patients [33]. Notably, the blunted BOLD activity in DMPFC under specific AM recall irrespective of memory valence in the rMDD group also would be consistent with a reduction in gray matter that persists across MDEs in MDD. That this region was more active in HR and HC relative to rMDD participants during both positive and negative specific AM recall suggests that HRs and HCs are able to regulate their emotional responses to both positive and negative AMs to a greater extent than rMDD participants. Additional support for this hypothesis comes from findings that reduced attention to emotional experiences (both positive and negative) is associated with better recovery from MDD [34].

In contrast, the precuneus was more active in HR and HC participants relative to rMDD participants during positive AM recall, while the opposite pattern was observed during negative AM recall. This region has been implicated in analytical self-referential processing and taking on a first person perspective [35,36]. Hemodynamic activity was reportedly increased in this region in MDD patients compared to controls during presentation of negatively valenced emotional faces [37], but decreased in MDD relative to HCs during specific AM recall matched for vividness and memory age [9]. The valence and group specific effects conceivably suggest that HR and HC participants direct attention inwardly during positive AM recall to a greater extent than rMDD participants, while rMDD participants direct attention more towards themselves during negative AM recall. However, the precuneus is also associated with specific relative to general AM retrieval [35], and our results could also be interpreted as evidence that rMDD participants have more difficulty recalling positive and less difficulty recalling negative AMs relative to the HR and HC participants, and that the a persistent marker of a previous MDE in reflected by the ease of recalling negative relative to positive AMs.

BOLD activity in the DLPFC was also increased in rMDD relative to HR and HC participants during negative AM recall. Increased BOLD activity in this region has been found when healthy individuals focus on their personal traits [38], during induced sadness [39], and while performing attentionally demanding cognitive tasks [23].

Our hypothesis that rMDD participants would have increased activity in brain regions implicated in self-referential processing and rumination was partially supported. rMDD participants indeed showed increased activity during negative AM recall in the precuneus and DLPFC, which are thought to play a role in ruminative processes. However, the majority of the differences were such that HR and HC participants had *increased* activity in regions involved in emotional regulation and in which increased activity correlates with better treatment response in depressed patients. These results suggest the hypothesis that HR participants are able to stop rumination and self-reflection and instead engage in emotional regulation strategies. The extent of rumination elicited by each memory were not examined in the current study, or in studies of AM recall in MDD in general, but future studies would benefit from incorporating these measures.

Interestingly, a study comparing adolescents with a first degree family relative with MDD to healthy controls on emotional regulation via positive AM recall found the HR group to have decreased activity in regions associated with the top-down regulation of emotion compared to controls [40], whereas we found that HR and HC participants recruit these regions to a similar extent. Because the mean age of onset for MDD is the mid-20s [41] and because the mean age in our HR group was early 30s, it is possible that our HR sample predominantly represented a resilient group beyond the age of peak susceptibility and that recruitment of regions involved in the top-down regulation of emotion to a similar or greater extent than HCs suggests recruitment of these regions promotes resiliency and serves an adaptive function in this high-risk population. Longitudinal designs which follow high-risk individuals as they progress from risk to disease state to remission would allow for direct testing of this hypothesis.

These results are intriguing when viewed in light of results from other studies within our laboratory examining AM recall in mood disorders, and allow us to directly test whether distinct at-risk populations differ from each other as referenced to low-risk controls. As in the current analysis, when HC, HR and currently depressed participants were compared to each other during specific AM recall, the majority of the differences were driven by the depressed group, with the HR and HC participants showing similar BOLD activity (except for the few regions highlighted in the introduction of the current manuscript), and further suggests our HR sample may be a resilient sample [5]. Interestingly, when comparing rMDD to HC and dMDD participants, in response to positive AM recall the mean BOLD activity increased in rMDD relative to dMDD participants in precuneus, DMPFC, and OFC [7]. In the current study, we found activity in these regions decreased in rMDD relative to HR and HC participants, implying that BOLD activity in these regions is intermediate in rMDD participants, between the dMDD and HR participants. These results implicate regions involved in the regulation of emotion as differentiating HR and rMDD participants, suggesting the hypothesis that a persistent mark of MDD with respect for AM recall may relate to altered emotional regulation. Experiencing a depressive episode may consequently reduce one’s ability to regulate emotional responses during AM recall, and previous research suggests that rumination is only related to depressive symptoms in the presence of maladaptive coping/regulation strategies [42].

Several limitations of the current study merit comment. Due to the nature of autobiographical memory recall, it is impossible to control the number of memories participants recalled in each mnemonic and valence category beyond the systematic use of cue words. Thus more specific AMs were recalled in the HCs relative to the other groups. Different bin sizes for each group in the fMRI analysis is a limitation inherent to imaging studies of episodic memory recall in participants with clinical pathology, and it is desirable and standard to include all usable trials in the analysis as opposed to removing trials from the HCs in order to match the number of bins between groups [5,6,7,43,44]. Nevertheless, the mixed model approach we applied for performing statistical comparisons across groups was robust to imbalances in the numbers of trials per bin across groups. Future studies could address this limitation by developing alternative methods for cueing AMs to elicit more balanced of specifically targeted AM types (*i.e.*, positive specific). Additionally, there are a rage of other factors not examined that may have influenced the behavioral and neuroimaging data and which may determine those individuals who are resilient to experiencing future depressive episodes and those who will experience a future major depressive episode (for example, rumination or previous experience with cognitive therapy). It is difficult, if not impossible, to take into account all of the factors. Nevertheless, future studies would benefit from adding additional measures such as rumination and previous treatment history.

## 4. Materials and Methods

### 4.1. Participants

We have previously published results using the HR and rMDD participant groups. However, these papers did not directly compare HR and rMDD samples. Instead, one paper examined neurophysiological correlates of AM recall for HR *versus* healthy control and currently depressed participants [5], while the other paper examined neurophysiological correlates of AM recall for rMDD *versus* healthy control and currently depressed participants [7]. Additionally, we have included an entirely new independent sample of HCs.

Sixty medically healthy, right-handed individuals ages 18–55 were evaluated for their eligibility to enter one of three groups: Psychiatrically healthy participants (*n* = 20), psychiatrically healthy participants with a first-degree relative with MDD (*n* = 20), and patients remitted from MDD as defined by DSM-IV-TR criteria plus a Hamilton Rating Scale for Depression (HDRS) score < 7 [45,46] (*n* = 20). Currently depressed individuals were not included in the current study to allow focus on vulnerability factors independent of current mood state. Volunteers, recruited from the community via advertisements, underwent medical and psychiatric screening evaluations at the Laureate Institute for Brain Research, which included the Structural Clinical Interview for *DSM-IV* disorders [47], and an unstructured diagnostic interview with a psychiatrist.

Exclusion criteria included current pregnancy, general MRI exclusions, serious suicidal ideation, psychosis, major medical or neurological disorders, exposure to any medication likely to influence cerebral function or blood flow within three weeks, and meeting *DSM-IV* criteria for drug/alcohol abuse within the previous one year or for alcohol/drug dependence (excepting nicotine) within the lifetime. Additional exclusion criteria applied to rMDD subjects were the presence of any depressive symptom severe enough to impair function or use of a psychotropic drug within three months [46]. While half of the remitted participants had never previously taken an antidepressant medication, 25% had previously taken 1–2 antidepressants and 25% had previously taken 3 or more antidepressants. The average time off antidepressants was seven years. Additional exclusion criteria applied to HR and HC participants were current or past history of any major psychiatric disorder, or a history of psychotropic medication use. After receiving a complete explanation of the study procedures, all participants provided written informed consent as approved by the Western IRB. Participants received financial compensation for their participation. Research was performed in compliance with the Code of Ethics of the World Medical Association, the Declaration of Helsinki, and the standards established by the Western IRB (Under Protocol 2010-004-01 initially approved 11/2010).

Intelligence testing was performed using the two-subtest version of the Wechsler Abbreviated Scale of Intelligence [48]. Anxiety and depressive symptoms were rated on the scanning day using the State-Trait Anxiety Inventory [49], the 21-item HRSD, with a score of less than 7, the cut-off for being in the non-depressed range [50], the Montgomery-Asberg Depression Rating Scale (MADRS), with a score of less than 6 considered to be in the non-depressed range [51], and the Profile of Mood States (POMS) [52]. With regards to the POMS and STAI, higher scores indicate worse overall mood.

### 4.2. Image Acquisition

BOLD fMRI was performed on a 3T GE Discovery MR750 scanner and eight-channel receive-only head coil. Gradient-recalled, echoplanar imaging (EPI) with sensitivity (SENSE) was used for fMRI with the following parameters: Repetition time (TR) = 2000 ms, echo time (TE) = 25 ms, SENSE acceleration = 2, flip angle = 90°, matrix = 96 × 96, field-of-view (FOV) = 24 cm, forty 2.9mm axial slices, voxel size = 3 × 2.5 × 2.9 mm^3^. A total of 211 EPI images were acquired in each of ten 7 min runs during the AM task. The first four images of each run were discarded to allow for steady-state tissue magnetization. High-resolution T1 weighted anatomical MRI scans (TR/TE = 5 ms/1.93 ms, flip angle = 8°, matrix = 256 × 256, FOV = 24 cm, slice thickness = 1.2 mm, 120 axial slices) also were acquired for co-registration with the EPI series.

### 4.3. fMRI Autobiographical Memory Task

In this event-related design, participants were presented 60 words (20 positive (e.g., success), 20 negative (e.g., danger), 20 neutral (e.g., journal)) using E-Prime software (Psychology Software Tools Inc., Sharpsburg, PA, USA). During fMRI, participants were presented with a cue word for 12 s and instructed to recall a past experience. Following the cue, participants rated the retrieved memory on specificity (specific, categorical, extended, semantic, repeat, no memory) according to the standard definitions used in the AM literature [1], and valence (negative, somewhat negative, neutral, somewhat positive, positive, no memory). Participants had 10 s to assign each rating. Prior to scanning we provided definitions and examples of each memory type, and ensured that the participants could provide two correct examples of each memory type before the fMRI task.

The AM recall condition was compared to a semantic example generation condition to control for abstract/general knowledge retrieval. Participants were presented with an example generation cue word for 12 s and instructed to think of at least seven examples from the presented category. Ten positive (e.g., flowers), 10 negative (e.g., villains), and 10 neutral categories (e.g., instruments) were presented. Following an example generation cue word, participants rated the ease with which they were able to generate examples (very easy, easy, somewhat easy, somewhat difficult, difficult, very difficult) and the number of examples they generated (0, 1–2, 3–4, 5–6, 7, 8 or more). Participants had 10s to select each rating.

Following the presentation of each cue and each set of ratings, participants engaged in a riser detection task as a control for visual input/attention. All example generation/memory cue words were scrambled into lowercase non-word letter strings and participants were instructed to count the number of risers in the string, defined as a letter with a part rising above the tops of the other letters (e.g., “gulmnh” has the risers l and h). The presentation of each letter string was jittered with an average presentation time of 6 s. For a randomly selected one-half of these strings, a 2 s period followed where participants selected whether the number of risers in the previous string was even or odd.

The order of memory and example cue word presentations was pseudo-randomized with restrictions on order presentation to prevent sequential presentations of a particular valence. Within each of ten runs, participants were presented with six memory cue words, three example generation cue words, and 18 riser letter strings in the order: Cue word-riser (1/2 followed by odd/even question)—ratings-riser (1/2 followed by odd/even question). Two computers time-linked to the image acquisition of the MRI scanner controlled stimulus presentation and behavioral response collection. Participants observed the stimuli using a mirror system attached to the head coil.

Following the scan, participants were presented with all AM cue words and asked to describe the memory for experimenter KY to corroborate participants’ specificity ratings. The experimenter was blind to diagnosis at the time of rating. In addition to standard memory categorizations of specific categorical, extended, and semantic [1,53], a memory was categorized as “can’t recall” if the participant was unable to recall the memory retrieved during fMRI, or if the reported memory was rated as a different categorization than the participant selected during the fMRI task. As can be seen in Table 1, the proportion of memories labeled as can’t recall was approximately 10% for each participant group indicating high reliability between participant and experimenter ratings. Additionally, an independent rater scored 40% of responses to establish inter-rater reliability (agreement = 91%). Also during the post-scan interview, participants rated each memory on arousal (5 point scale ranging from not at all to highly arousing), vividness (5 points scale ranging from not at all to perfectly clear and vivid), and when the memory occurred (childhood, adolescence, adulthood > 1 year ago, between 6 months and 1 year ago, and < 6 months ago).

### 4.4. Assessment of Behavioral Performance during fMRI

Behavioral data were analyzed using SYSTAT 13 (Systat Software Inc., San Jose, CA, USA). Potential group differences in age, IQ, anxiety ratings, performance on the riser detection and example generation control tasks, and the percent of memories recalled at each specificity level (specific, categorical, extended, semantic, no memory, and can’t recall post-scan) were assessed using an Analysis of Variance.

The *a priori* hypothesis testing focused on the properties of the *specific* and *categorical* memories, as these are the autobiographical memory classifications in which MDDs and HCs differ [1]. To increase power, the following variables were collapsed: Low and very low arousal were considered together to generate a low arousal variable, high and very high arousal to produce a high arousal variable, low and very low vividness to produce a low vividness variable, high and very high vividness to create a high vividness variable, somewhat positive and positive to create a positive variable, and somewhat negative and negative to create a negative variable. Repeated measures ANOVAs were performed for the between subjects variable Diagnosis (HC, HR, rMDD), and the repeated measures of Type (Specific, Categorical) and either Valence (positive, negative, neutral), Arousal (low, medium, high), Vividness (low, medium, high), or Age (childhood, adolescence, after 18 but longer than 1 year prior to scan, between 6 months and one year prior to scan, and less than 6 months before scan) for the dependent variable Percent of Memories recalled. The threshold criterion for significance was set at *p* < 0.05, corrected for multiple comparisons using the Bonferroni correction.

### 4.5. fMRI Processing and Analysis

Image pre-processing and analysis were performed using AFNI [54]. Image pre-processing consisted of slice acquisition time correction, within-subject realignment, co-registration between the anatomical and functional images, spatial normalization to the stereotaxic array of Talairach and Tournoux [15], and smoothing using a 4mm full-width at half-maximum Gaussian kernel. Using 3dDeconvolve for each participant, the hemodynamic response to each event type was modeled as a boxcar function convolved with a cannonical hemodynamic response function. Regressors modeling the task and motion parameters were used in the model. The main effects-of-interest were the cue word presentations that prompted *specific* memory recall, *categorical* memory recall, and example generation. In addition to regressors modeling the main effects, each design matrix included regressors modeling rating selection, cue presentation where the retrieved memory was not recalled, cue presentation where other types of memories were recalled (extended, semantic), and even/odd riser question presentations. The non-word letter strings used as stimuli for the riser detection task were modeled as the baseline.

At the group level, mixed-effects 3dANOVAs were used to identify regional differences in the blood-oxygen level dependent (BOLD) signal between rMDD, HR, and HC participants for the following comparisons: Specific Memories *versus* Example Generation, Categorical Memories *versus* Example Generation, and Example Generation *versus* Riser Baseline. Because the valence of recalled specific AMs are often reported to differ between HCs and rMDDs [5,55,56], and because we found differences between HCs and HRs and rMDDs in the percent of specific positive and negative (but not differently valenced categorical) AMs, the additional contrasts of Specific Positive Memories *versus* Positive Example Generation and Specific Negative Memories *versus* Negative Example Generation also were performed. Additional one-sample *t*-tests were performed using 3dMEMA to compare the BOLD response between the Specific Memories *versus* Example Generation condition, the Categorical Memories *versus* Example Generation condition, and Example Generation *versus* the Riser Baseline condition for all groups combined (all subjects pooled together). The significance criterion for detecting differences was set at *p*_corrected_ < 0.05 determined using 3dClustSim (cluster size > 30 voxels, thresholded at voxel *p* < 0.001 with a smoothing kernel of 5 mm).

## 5. Conclusions

The current study demonstrates that the experience of depression results in altered patterns of neural activity during AM recall which are not present prior to illness onset in those at risk, and which persist into remission of depressive symptoms. The HR participants engaged regions previously implicated in emotional regulation and attentional focus on others to a greater extent than the rMDDs, while rMDDs engaged regions implicated in self-referential processing during negative AM recall to a greater extent than HRs. As previous studies have found that interventions for MDD which focus on disengagement from analytical self-focus/rumination increase AM specificity [57], and reduce relapse rates [58], the results of the current study suggest that it may be beneficial not only to target enhancing emotional control during AM recall and disengagement from analytical self-focus on negative AMs but also to promote analytic self-focus on positive AMs.

## Supplementary Files

Supplementary File 1
