# Supplementary material for: Functional Neuroimaging Correlates of Autobiographical Memory Deficits in Subjects at Risk for Depression
Source: Brain Sci. 2015 Apr 24;5(2):144–64. doi: 10.3390/brainsci5020144 (PMC4493461; doi:10.3390/brainsci5020144)
Supplement: Supplementary File 1 [file brainsci-05-00144-s001.pdf]

Supplementary Information

Table S1. Properties of specific and categorical AMs for each participant group #.

Valence		HC	HR	rMDD
Specific AMs	Positive	63.0 (14.2)	54.5 (11.2) *	50.8 (11.4) *
	Negative	26.3 (13.2)	33.9 (10.7) *	35.9 (13.7) *
	Neutral	10.7 (7.71)	11.6 (8.81)	13.3 (13.2)
Categorical AMs	Positive	58.7 (17.1)	59.3 (15.2)	55.7 (12.6)
	Negative	23.5 (13.2)	24.7 (10.8)	26.2 (12.9)
	Neutral	17.8 (16.6)	16.0 (15.3)	18.1 (10.7)
**Arousal**				
Specific AMs	Low	33.2 (23.9)	32.0 (21.8)	31.9 (22.3)
	Medium	30.8 (12.5)	30.1 (14.5)	28.7 (14.4)
	High	36.0 (24.9)	37.9 (21.8)	39.4 (24.9)
Categorical AMs	Low	34.9 (28.3)	36.4 (26.2)	32.4 (23.7)
	Medium	31.3 (16.2)	31.9 (22.0)	30.9 (17.4)
	High	33.8 (28.2)	31.7 (25.9)	36.7 (25.3)
**Vividness**				
Specific AMs	Low	10.2 (12.5)	11.3 (10.9)	15.4 (16.6)
	Medium	23.1 (12.4)	28.3 (14.1)	21.4 (12.1)
	High	66.9 (21.1)	60.4 (18.0)	63.2 (23.7)
Categorical AMs	Low	23.1 (20.6)	24.7 (25.9)	24.5 (18.9)
	Medium	38.6 (19.3)	35.7 (18.0)	37.9 (17.2)
	High	38.3 (23.5)	39.6 (22.6)	37.6 (25.4)
**Age When Memory Occurred**				
Specific AMs	Childhood	11.6 (9.02)	11.6 (8.79)	10.1 (8.32)
	Adolescence	14.8 (9.98)	12.5 (10.0)	17.6 (14.7)
	Remote Adult	29.9 (18.9)	33.2 (24.6)	29.5 (19.5)
	Adult: 6mo-1yr	12.9 (8.24)	11.0 (7.07)	8.46 (8.75)
	Recent Adult	31.0 (13.3)	31.7 (17.6)	34.6 (18.1)
Categorical AMs	Childhood	14.6 (15.6)	17.2 (15.4)	11.3 (11.4)
	Adolescence	19.0 (16.3)	12.9 (14.0)	18.5 (14.2)
	Remote Adult	18.9 (16.0)	22.1 (17.3)	23.2 (18.6)
	Adult: 6mo-1yr	4.95 (7.03)	8.67 (10.0)	6.75 (6.31)
	Recent Adult	42.6 (22.9)	39.1 (19.2)	40.2 (17.9)

Abbreviations: AM = autobiographical memory; HC = healthy control; HR = high-risk; rMDD = remitted major depressive disorder. # Note that the values indicate the percent of memories recalled within each subcategory and numbers should add up to 100% for specific and categorical memories separately for each property (valence, arousal, vividness, memory age); * Indicates a significant difference from the HC group at $p < 0.05$.
